# The Neuroimaging Studies in Children with Ventriculoperitoneal Shunt Complications: A 10 Years Descriptive Study in Tehran

**DOI:** 10.2174/1874440001812010001

**Published:** 2018-01-22

**Authors:** Mohammad Vafaee Shahi, Samileh Noorbakhsh, Vida Zarrabi, Banafsheh Nourozi, Leila Tahernia

**Affiliations:** 1 Pediatric Department, Pediatric growth and development research center, Institute of Endocrinology and Metabolism, University of Medical Sciences, Tehran, Iran; 2 Department of pediatric infectious diseases , University of Medical Sciences. Tehran, Iran; 3 Department of Radiology University of Medical Sciences, Tehran, Iran; 4 Department of Pediatric, University of Medical Sciences, Tehran, Iran; 5 Department of Pediatric, Tehran University of Medical Sciences, Tehran, Iran

**Keywords:** Brain CT scan, Cerebrospinal fluid, Children, Ventriculo peritoneal shunt, Neuroimaging, hydrocephalus

## Abstract

**Background:**

Any mismatch between the production and absorption of CSF results in hydrocephalus. In most cases, the selected choice of treatment is the ventriculoperitoneal shunt insertion. Although, the surgery could have complications such as infection, shunt malfunction, subdural hematoma, seizure and Shunt immigration; so, the early and proper detection of these complications could result in better prognosis. The aim of this study was to evaluate and compare the efficacy of CT scan, CSF analysis and X-ray radiography in detection of shunt complications and problems in shunt placement and further follow-up in hospitalized children.

**Methods:**

The medical records of children in Rasul Akram hospital in Tehran were reviewed retrospectively in the last 10 years, from 2006 to 2016. All data were recorded in the prepared form including the age, sex, shunt complication, CT scan and CSF characteristics.

**Results:**

The total number of 95 patients were interfered in this study including 56 males (58.9%) and 39 females (41.1%). The mean age at the onset of complications were 2.8±2.2 years-old. The shunt obstruction (60%) and infection (25.3%) were the most common complications. The CT scan was able to detect 36.5% of shunt complications. The CT scan had the sensitivity and specificity of 50 and 87%, respectively in detection of shunt obstruction. The all cases of brain hematoma and hemorrhage were revealed by CT scan. On the other hand, the CT scan had 20% of sensitivity and 60% of specificity in the detection of shunt infection. The CSF evaluation in shunt infection revealed 92% hypoglycemia, 87.5% pleocytosis, and 62.5% positive CSF culture. CSF had the sensitivity, specificity, positive predictive value and negative predictive value of 92, 82, 63 and 97%, respectively. The patient's symptoms and signs were helpful in obtaining higher test accuracy.

**Conclusion:**

The CT scan was not a good sensitive and specific study in the detection of shunt obstruction and infection, but it was very accurate in detection of hemorrhage and hematoma. On the other hand, CSF evaluation was a reliable test in shunt infection disclosure.

## 1 INTRODUCTION

Ventriculoperitoneal shunt treats hydrocephalus by altering CSF flow within the brain. In most of cases, shuns consist of three parts that are serially connected to each other: the proximal catheter, one-way valve and distal catheter [1].According to published statistics, around 40000 CSF shunts are implanted annually in USA [2]. But, treatment failure with this method is estimated about 40% during the first year of shunt insertion [3]. The shunt complication diagnosis is initially suspected according to the history and physical examination and high intracranial pressure; however, imaging study is often performed to confirm the diagnosis and reveals the underlying cause. So, radiologists are required to be familiar with the radiologic manifestations of shunt complications.

Shunt complications can be divided into three general categories: mechanical failure (improper insertion of shunt, partial shunt blockage, fracture or displacement of the shunt portions or shunt movement from the initial place), dysfunctional shunts and infections [4]. Infection as the major cause of shunt malfunction and related complications [5-15] occurs between 8 to 12%. Shunt infection often occurs during the first 6 months of shunt placement mostly resulted from contamination with normal skin bacteria flora during surgery [16]. But sometimes it occurs several months after surgery that probably is due to infection in a distant location urinary infection [15-17]. In admitted children in neurosurgical wards in academic hospitals, yearly, a large number of shunts are being placed for various reasons [17, 18]. Shunt complications are reported in some cases with emergent shunt insertion [19-21]. Accordingly, in this study we were to emphasize on the importance of early diagnosis of shunt complications and immediate management. The aim of this study was to evaluate and compare the efficacy of CT scan, CSF analysis and X-ray radiography in detection of shunt complications and problems in shunt placement and further follow-up in hospitalized children.

## 2 METHODS

The total number of children with hydrocephalus treated with shunt insertion by standard technique in Rasul Akram hospital were 200 patients during 10 years from 2006 to 2016. This retrospective descriptive study reported shunt complications in 95 children from total admitted patients in Rasoul Akram Hospital during past 10 years. Information related to shunt complications were extracted and inserted in related forms. Brain CT scan, X-ray radiography and CSF analysis findings associated with selected patients, gathering in hospital information system, were revised by executive radiologist. CSF analysis data were extracted from previous patients records in order to compare information before and after surgery with data obtained before and after shunt complications. Other data related to the etiology, symptoms and complications of shunt insertion were collected in data forms. This project adheres to the principles of Helsinki treaty and all obtained information from medical records remained confidential. Samples were selected from children's clinical records in the archives of Rasoul Akram Hospital during the last 10 years. Sample size was determined based on previous sampling according to the total number of hospitalized children with shunt complications. Our estimated sample size was around 80 patients. But during the project, we tried to enter the highest number of patients. Inclusion criteria included patients between 0 to 18 years-old, shunt placement indication for any reasons, complete radiological and laboratory data records, presence of at least one shunt complication. Exclusion criteria included age above 18 years old, no final diagnosis of shunt complication, incomplete patient records.Data were analyzed by SPSS statistical software (version 13.5). Frequency, central statistical indices, such as mean and dispersion indices like standard deviation were determined for all patients. Chi-square and T-test were used in order to compare shunt complications in different groups of patients. In this study, statistically significant level was considered to be 0.05.

## 3 RESULTS

This study was conducted on 95 patients including 56 males (58.9%) and 39 females (41.1%). The average age of patients was 27+- 5 days at the time of shunt placement. The average age at the onset of shunt complications was 2.8 + - 2.2 years. All children were hospitalized with the diagnosis of shunt complication and were evaluated through radiographic and laboratory tests.

Shunt complications were classified according to Table **1** into five categories including obstruction, fracture and detachment and displacement, abnormal flow of CSF, hematoma and bleeding and finally infection. In our study, the most common shunt complication was shunt occlusion with 60% incidence. Shunt infection was considered in more than 25% of complicated shunts. The abnormal CSF flow, subdural hematoma and hemorrhage were considered as the least incidence in shunt complications.

Serial plain radiography and CT scan were conducted in all studied children in order to check the shunt status. CT scan findings were analyzed on the basis of (Table **2**) Children without CT scan performed at the onset of shunt complication were excluded from this study because we were looking for changes in CT scan at the time of shunt insertion and the onset of shunt complication. Normal CT scan and ventricular enlargement were the most frequent findings in documented reports by radiologists. CT scan findings were evaluated on the basis of age. There was not a statistically significant relationship between CT scan findings and the average age of patients.

Normal CT scan findings detected more in males than females. However, there was no significant difference in the findings of CT scans in children with shunt complications based on gender (Table **3**).

The majority of CT scans with normal findings were related to patients with shunt insertion indications of congenital hydrocephalus and intra-ventricular hemorrhage. Newly developed ventricular enlargement in CT scans were reported in 53% and 17.6% of patients with congenital hydrocephalus and CNS infection shunt indications. The difference in CT scan findings by the type of hydrocephalus was not statistically significant (Table **4**).

Obstruction was reported in majority of the cases with normal CT findings and ventricular enlargement, respectively. Fracture, displacement, shunt separation, hematoma and hemorrhage were observed in CT scan findings. Normal CT scan findings were reported in about a quarter of infectious shunt complications, but three quarters were detected with ventricular enlargement and abscess in CT scan. According to the conducted analysis, there was a significant relationship between CT scan findings and shunt complications (Table **5**).

The most common clinical signs included restlessness and irritability, following by nausea and vomiting. Headache, growth retardation and loss of appetite were other common symptoms in children with shunt complications (Table **6**).

The shunt occlusion was not often associated with pathologic findings in CSF analysis. Only 5.3% of patients with shunt occlusion were associated with hypoglycemia, CSF pleocytosis and positive culture in 5.3%, 7% and 5.3%, respectively. All patients with shunt fracture had normal CSF findings. On the other hand, only one positive (25%) CSF culture was reported from abnormal CSF flow. Abnormal CSF was present in 66% of cases with hematoma and hemorrhage. Also, 8.3% of infected shunts had normal CSF analysis (Table **7**).

The most common detected organism was Coagulase-negative Staphylococcus, found on CSF culture results. Staphylococcus aureus was the second most common bacteria. The least frequent bacteria were gram-negative bacilli and Streptococci. No poly-microbial CSF infection was reported in this study (Table **8**).

The average time of complication occurrence due to occlusion was 35+ _ 12.5 days. However, fracture and shunt displacement occurred mostly one year after insertion time. Hematoma and hemorrhage appeared averagely after 25 +_ 22 days and infection occurred in 6 months following shunt placement.

CT scan findings had a significant correlation with the time duration between shunt insertion and its complications (Table **9**).

## 4 DISCUSSION

In our study, the most common shunt complication was shunt obstruction (60%) following by shunt infection (23.3%). This result was similar to the results of other previous studies. In Caldarelli study in 1996, shunt complications were evaluated in the first postoperative year in 170 children with meningomyelocele. In this study, the authors realized that 45.9% of the patients presented one shunt malfunction (75% with mechanical causes and 25% due to infection) [6, 24-28]. Congenital hydrocephalus and IVH were the most common shunt insertion etiologies. This was also similar to the results of previous studies [1-3].

In a randomized controlled trial by Piatt in 2008, VP shunt failure diagnosis in children with hydrocephalus was considered based on clinical symptoms and observations at scheduled follow-up visits 3 months, 1,2 and 3 years following shunt insertion. In this study, 38% were reported with shunt failure. The most common symptoms and signs strongly associated with shunt failure were bulging fontanel, fluid collection along the shunt, depressed level of consciousness, irritability, abdominal pain, nausea and vomiting, accelerated head growth and headache. Fever was observed specifically in shunt infection [29]. In another study by Hugh.J *et al*. in 2001, the most common symptoms were nausea and vomiting, irritability, decreased Level Of Consciousness (LOC), erythema, and bulging fontanelle. Between 9 months and 2 years after shunt insertion, only loss of developmental milestones and decreased LOC were strongly associated with shunt failure. However, the absence of a symptom or sign was associated with chance of shunt failure between 9 to 29% [30]. But in the present study, the most common clinical symptoms were restlessness and agitation followed by nausea and vomiting. Other common symptoms in children with shunt complication included irritability, loss of appetite and growth retardation. However, none of these clinical signs and symptoms are sensitive enough to rule out shunt malfunction [29].

The most commonly used imaging modality to evaluate shunt malfunction is CT scan. New enlarged ventricles are the most frequent shunt obstruction feature. Other CT scan findings included: the cortical sulci effacement, loss of the basal cisterns and periventricular edema due to transependymal CSF absorption [31]. CT scan has between 53% and 92% sensitivity for shunt malfunction detection [31-34]. In the present study, in majority of the cases, shunt occlusion was associated with normal CT findings followed by ventricular enlargement in second rank. Results sensitivity and specificity were 50% and 87%, respectively. Consequently, CT scan was not a sensitive test to evaluate suspected shunt blockage. In a retrospective study of 174 adults with shunt malfunction CT had a sensitivity of only 52%, a specificity of 78% and negative predictive value of 88% for shunt malfunction [35]. This study only included patients who had had shunt series performed, so it may have underestimated the sensitivity of CT by excluding patients who were evaluated with CT alone.

On the other hand, CT scan sensitivity and specificity for fracture detection were 43% and 100% respectively. This suggested that if a fracture was suspected in serial plain radiography, CT scan is helpful to confirm the diagnosis. CT scan could show all cases of increase or decrease in CSF flow in the form of edema. Moreover, hematoma and hemorrhage were diagnosed with high sensitivity and specificity in CT scan images. It demonstrated the effective role of CT scan in diagnosis of suspected hemorrhage and hematoma.

Statistical analysis demonstrated that CT scan findings in complicated shunts were not sex-dependent, but significant association with patients' age was obvious; for example, although the average age of patients with normal CT findings was around 3.6 +- 1.9 years, but the average age of patients with ventricular enlargement and hemorrhage were 1.8 +- 1.7 years and 5.3+- 1.5 years, respectively. Probably, attention to the age, regardless of gender, was helpful in diagnosis of shunt complications. Altogether, CT scan was able to detect only 58 patients from total 95 cases with proven diagnosis of shunt complications (61% sensitivity). In conclusion, it seems that CT scan alone is not a reliable modality for investigation of suspected cases of shunt complications. Probably; it is better to use combination of CT scan, history, physical exam and other diagnostic modalities in order to increase diagnostic accuracy. In a study by Pitetti *et al.* [23], suspected cases of shunt malfunction were evaluated in emergency department. It showed that routine plain radiography in evaluation of suspected shunt malfunction in children was beneficial because some patients may have abnormal plain radiography despite normal CT scan findings.These results were against Karppinen *et al.* [22] study results, that believed in more effective role of CT scan compared to serial X-rays. Shunt series radiographs were used to detect mechanical shunt defects, such as shunt discontinuity or kinking. In both children [4, 7] and adults [33] studies sensitivity of radiographic shunt series was very low. Shunt series rarely (1-2%) detect complications not identifiable by initial CT scan. Therefore, shunt series are still indicated in the evaluation of potential shunt malfunction [34].In our results, CSF examination of shunt could identify 48 cases of shunt complications in total 95 patients. In this study, CSF pressure measurement was not used in evaluation of abnormal cases, however using this criterion did not seem logical in evaluation of shunt complication due to various etiologies of shunt insertion and different types of shunt devices. If the opening or closing CSF pressure were monitored, more cases of shunt complication, particularly shunt occlusion, would be detectable. Infection was not expected to be recognized properly by CT scan images. Ventricular enlargement was reported in only 14.7% of diagnosed shunts with infection on CT scan. But all cases of abscess were obvious on CT scan.Regardless of patients with abscess on CT scan, 20% sensitivity and 60% specificity proved that this modality is not desirable for evaluation of suspected shunt infection. As already was expected in CSF examination, hypoglycemia was obvious in infected shunts. In a new study by Hongri Z *et al*. in 2017, A total of 14 participants consisting of 7 patients with suspected shunt malfunction and 7 control cases with apparent normal drainage, 0.1 mL of 5% glucose solution was injected into the reservoir and 0.1 mL of cerebrospinal fluid was withdrawn from the reservoir 20 minutes later to measure glucose concentration. They resulted that the glucose concentration in cerebrospinal fluid of the shunt malfunction group was greater than that of the control group [35].

Since the main causes of shunt infection are bacterial and fungal, CSF hypoglycemia was not unexpected (culture results were all in favor of bacterial infection).Pleocytosis was reported in 87% of patients with infected shunt which was predictable given the major pathogens. Positive smear was reported in 75% and positive culture may have been due to specific sampling or need for special culture media. About 6% of shunt occlusion cases were associated with positive CSF findings. Positive culture could be due to contamination of samples and hypoglycemia could be a probable result of underlying pathogen or simultaneous shunt infection. One case of abnormal CSF flow was also associated with positive culture which was likely due to sample contamination. In general, sensitivity and specificity of CSF evaluation in detection of shunt infection was about 92% and 82%, respectively. However, CSF positive and negative predictive values were 63% and 97%. This means that in case of negative CSF evaluation, shunt infection could be rejected with high accuracy. In a study by Caldarelli *et al.* [24] the worth of abnormal CSF analysis was noted in occurrence of infectious complications. Our findings contradicted Fulkerson *et al.* study results [25]. They did not believe in effective role of CSF evaluation in diagnosis of shunt failure. Although their study was performed on premature newborns, it was probable that difference could be due to the nature of CSF in newborns. Results of a study in Sweden [26] were also significant because it showed that CSF examination could be normal in presence of shunt blockage. Although the study focused on shunt infection, CSF changes due to shunt occlusion were justified reactively. These results were considerably similar to findings of a study in Japan. In this study [27], early CSF changes resulted in shunt placement was considered a normal reaction, not pathological. So, it seems that in cases of early onset shunt failure, CSF examination should be analyzed more accurately. This point also was mentioned in a study conducted in USA [28].The sample size in this study was appropriate. All patients had a proven diagnosis of shunt related complications. There were no cases with a suspected or underestimated diagnosis. Patients without radiological investigation like CT scan or lumbar puncture analysis were excluded from our study. CT scan images were reviewed by expert radiologists who helped us to obtain more reliable results. Another positive aspect of this study was simultaneous evaluation of radiologic and laboratory results which increased the accuracy of this study. One limitation of this study was to involve other beneficial and routine tests in evaluation of patients. All patients entered in this study had the proven diagnosis of shunt complications and complete laboratory and radiological studies. This could reduce a little the accuracy of the study.

## CONCLUSION

The results of this study on CSF changes and CT scan results in children with shunt complications demonstrated that CT scan was not able to provide great degree of sensitivity and specificity in detection of shunt infection and occlusion. On the other hand, CT scan was really sensitive in detection of brain hemorrhage and hematoma. CSF analysis had a high value in detection of shunt infection. Signs and symptoms of patients would increase the accuracy of diagnostic tests. No single clinical exam finding or imaging study is sufficient to rule out shunt malfunction. Clinical management should take into account history, clinical signs and symptoms, diagnostic information and finally neurological assessment.

## Figures and Tables

**Table 1:** Distribution of shunt complications in studied children.

Incidence %	Number of Incidence	Shunt complication
60	57	Obstruction
7.4	7	Fracture/Detachment/Displacement
4.2	4	Abnormal flow of CSF
3.15	3	Hematoma/Bleeding
25.3	24	Infection
**100**	95	Total

**Table 2:** Distribution of CT scan findings in children with VP-shunt complication.

**Distribution of CT scan findings in children with VP-shunt complication**			
*P* value	Frequency (%)	Mean age of patient	CT Finding
0.04	36.8) %)35	1.9±3.6	Normal
	(2.1%)2	2.8±2.8	No change
	(3.2%)3	1.7± 2.5	Fracture or Displacement
	(%35.8)34	1.7±1.7	Ventricular enlargement
	(3.2%)3	5.1±5.3	Bleeding
	(3.2%)3	1.5±2.6	Pneumocephalus
	10(10.5%)	1.2±2.5	Abscess

**Table 3:** Distribution of CT scan findings in children with shunt complications based on sex distribution.

*P* Value	Female (%)	Male (%)	CT Finding
0.9	14 (40%)	60%))21	Normal
	(50%)1	50%))1	No change
	1(33.3%)	2(66.7%)	Fracture or Displacement
	13(38.2%)	21(61.8%)	Ventricular enlargement
	1(33.3%)	2(66.7%)	Bleeding
	2(66.7%)	1(33.3%)	Pneumocephalus
	5(50%)	5(50%)	Abscess
	(%40)2	(%60)3	Periventricular and interstitial edema
	(%39(39.4	(%58.9)56	Total

**Table 4:** Distribution of CT scan findings in children with VP shunt complications by the type of hydrocephalus (*P*= 0.37).

Cyst and Tumor	Congenital	IVH	Spina bifida	CNS Infection	CT Finding
3(8.8%)	12(34.3%)	12(34.3%)	6(17.6%)	2 (5.7%)	Normal
0	9(45%)	7(35%)	1(50%)	1(50%)	No change
0	1(33.3%)	1(33.3%)	0	1(33.3%)	Fracture or Displacement
3(8.8%)	6(18.3%)	2(6%)	5 (17.4%)	6(17.6%)	Ventricular enlargement
0	2(66.7%)	1(33.3%)	0	0	Bleeding
0	3(30%)	0	0	0	Pneumocephalus
0	3(30%)	5(50%)	2(20%)	0	Abscess
0	3 (60%)	2(40%)	0	0	Periventricular and interstitial edema
6	42	23	14	10	Total

**Table 5:** (P<0.001) Distribution of CT scan findings in children with ventricular shunt.

Infection	Obstruction	Fracture/Detachment/Displacement	Abnormal CSF flow	Hematoma/Bleeding	CT Finding
**8(23%)**	**27(77%)**	0	0	0	Normal
1(50%)	0	1(50%)	0	0	No change
0	0	3(100%)	0	0	Fracture/ Displacement
5(14.7%)	29(85.3%)	0	0	0	Ventricular enlargement
0	0	0	0	3(100%)	Bleeding
0	3(100%)	0	0	0	Pneumacephalus
10 (100%)	0	0	0	0	Abscess
0	1 (20%)	0	4 (80%)	0	Periventricular/interstitial edema

**Table 6:** Distribution of CT scan findings in children with ventricular shunt complication based on patient 's symptoms.

Growth Failure	Restlessness/Irritability	Nausea/ Vomiting	Headache	Decreased appetite	CT Finding
6(17.1%)	21(60%)	22(63%)	12(34.3%)	15(43%)	Normal
0	1(50%)	1(50%)	1(50%)	0	No change
0	3(100%)	2(66.7%)	2(66.7%)	0	Fracture/Displacement
21(61.8%)	32(94%)	25(73.5%)	20(58.8%)	16(47%)	Ventricular enlargement
1(33.3%)	3(100%)	2(66.7%)	2(66.7%)	0	Bleeding
0	3(100%)	2(66.7%)	1(33.3%)	0	Pneumocephalus
0	5(50%)	5(50%)	5(50%)	5(50%)	Abscess
0	5(100%)	5(100%)	5(100%)	0	Periventricular/Interstitial edema
28(29.50%)	73(76.8%)	64(67.3)	48(50%)	38(38%)	Total

**Table 7:** Distribution of CSF findings in children with ventricular shunt complication based on the type of shunt complication.

Infection	Obstruction	Fracture/ Detachment/ Displacement	Abnormal CSF flow	Hematoma/ Bleeding	CSF Finding
2 (8.3%)	47 (82.5%)	7(100%)	3(75%)	1(33.3)	Normal
22 (92%)	3(5.3%)	0	0	2 (66.7%)	Hypoglycemia
21(87.5%)	4 (7%)	0	0	1 (33.1%)	CSF pleocytosis
18 (75%)	3(5.3%)	0	1 (25%)	0	Positive smear
16(62.5%)	3(5.3%)	0	1 (25%)	0	Positive culture

**Table 8:** Distribution of microbial etiology based on culture results.

Frequency %	Frequency	Bacteria
63	12	Coagulase negative staphylococcus
21	4	Staphylococcus aureus
10.5	2	Gram negative bacilli
5.3	1	Streptococcus

**Table 9:** Distribution of the average time of shunt complication occurrence after insertion of shunt.

P value	Average Time of Shunt Complication (Day)	Shunt Complication
0.0001	12.5±35	Obstruction
	64±38.2	Fracture/ Detachment/ Displacement
	53±124	Abnormal CSF flow
	22±25	Hematoma/ Bleeding
	62±150	Infection
